# Data set for the mass spectrometry based exoproteome analysis of *Aspergillus flavus* isolates

**DOI:** 10.1016/j.dib.2014.12.001

**Published:** 2014-12-15

**Authors:** Ramu Muthu Selvam, Rathnavel Nithya, Palraj Narmatha Devi, R.S. Bhuvana Shree, Murugesan Valar Nila, Naveen Luke Demonte, Chitra Thangavel, Jayapal Jeya Maheshwari, Prajna Lalitha, Namperumalsamy Venkatesh Prajna, Kuppamuthu Dharmalingam

**Affiliations:** a Proteomics Department, Aravind Medical Research Foundation, Dr. G. Venkataswamy Eye Research Institute, Aravind Eye Care System, Madurai, Tamil Nadu, India; b Department Of Ocular Microbiology, Aravind Eye Hospital, Aravind Eye Care System, Madurai, Tamil Nadu, India; c Cornea Clinic, Aravind Eye Hospital, Aravind Eye Care System, Madurai, Tamil Nadu, India

## Abstract

*Aspergillus flavus* is one of the predominant causative organisms of mycotic keratitis in tropical parts of the world. Extracellular proteins are the earliest proteins that come in contact with the host and have a role in the infection process. Exoproteins of *A. flavus* isolated from infected cornea, sputum and a saprophyte were pooled and identified using high resolution mass spectrometry in order to get the total exoproteome from cultures isolated from different sources. A total of 637 proteins was identified from the pooled *A. flavus* exoproteome. Analysis based on GO annotations of the 637 identified proteins revealed that hydrolases form the predominant class of proteins in the exoproteome. Interestingly, a greater proportion of the exoproteins seem to be secreted through the non-classical pathways. This data represent the first in-depth analysis of the representative *A. flavus* exoproteome of a large set of isolates from distinct sources. This data have been deposited to the ProteomeXchange with identifier PXD001296.

**Specifications table**

**Table:** 

Subject area	Biology, Mycology, Mycotic keratitis
More specific subject area	Exoproteomics of *Aspergillus flavus*
Type of data	List of identified proteins (.xlsx) and figures
How data was acquired	Tandem mass spectrometry ( LC-MS/MS) using Thermo Easy nLC 1000 (Thermo, USA) coupled to Orbitrap Velos Pro mass spectrometer (Thermo, USA)
Data format	Raw, analyzed and filtered
Experimental factors	N/A
Experimental features	Exoproteins prepared from the saprophyte (ATCC26), a sputum isolate and five corneal isolates of *A. flavus* were pooled and pre-fractionated on 1D SDS-PAGE. Proteins in the gel pieces were processed, subjected to tryptic digestion and the extracted peptides after the cleanup was analyzed in a high resolution mass spectrometer.
Data source location	Aravind Medical Research Foundation, Madurai, India.
Data accessibility	The mass spectrometry proteomics data have been deposited to the ProteomeXchange Consortium via the PRIDE partner repository with the dataset identifier PXD001296

**Value of the data**

• In-depth profile of *A. flavus* exoproteome
• Label-free quantitation of exoproteins reflecting their abundance in extracellular space
• Insight into the functions of the exoproteome
• Identification of probable infection related proteins
• A comprehensive protein set of different isolates from different niches.

## 1 Experimental design, materials and methods

### 1.1 Preparation of samples for mass spectrometry

Pooled exoproteins (hundred micrograms) were separated on a 10% 1D SDS-PAGE and stained with colloidal coomassie stain. Twenty-two bands were cut from the entire lane for processing. The gel band was cut into 1 mm^2^ pieces and were washed twice with water. Gel pieces were completely destained by repeated incubation in 25 mM ammonium bicarbonate prepared in 50% acetonitrile, followed by incubation in 100% acetonitrile to dehydrate the gel pieces. Dehydrated gel pieces were dried under vacuum and were rehydrated for 30 min on ice with 600 ng of trypsin (Invitrogen) in 5 µl of 100 mM ammonium bicarbonate in 10% acetonitrile. The gel pieces were overlaid with 20 µl of 40 mM ammonium bicarbonate in 10% acetonitrile and incubated at 37 °C for 16 h. Peptides were then extracted from gel pieces using 25 µl of 0.1% trifluoroacetic acid (TFA) in 60% acetonitrile and then with 20 µl of 100% acetonitrile. Extracted peptides were vacuum dried, desalted using C18 tips [1], dried and stored at 4 °C. Dried tryptic peptides were suspended in 10 µl of 0.1% formic acid (FA) and analyzed using LC-MS/MS.

## 2 Instrumentation

Tryptic peptides were pooled (Supplementary Table 1) and analyzed using a Thermo Easy nLC 1000 (Thermo, USA) coupled to Orbitrap Velos Pro mass spectrometer (Thermo, USA). A capillary RSLC column (EASY-spray column pepMap^®^RSLC, C18, 2 μm, 100 Å, 75 μm×50 cm or 15 cm Thermo SCIENTIFIC, CA) was used for separation of peptides. Samples were first loaded onto a pre-column (Acclaim^®^ PepMap 100, C18, 3 μm particle size, 100 Å, 75 μm×2 cm Thermo SCIENTIFIC, CA) from an autosampler at a maximum pressure of 700 bar. Following the pre-column, the tryptic peptides were analyzed using an analytical column with a linear gradient programme where the component of solution B (95% ACN in 0.1% FA) was changed from 5% to 100% over 90 min at a constant flow rate of 200 nL/min (from 5% to 30% over 72 min, 30% to 100% over 10 min, and kept at 100% for 5 min at a flow rate of 200 nL/min). The samples were acquired in positive mode electrospray ionization, with an ion spray voltage of 2.4 kV, capillary temperature of 200 °C, RF lens voltage of 69 and maximum injection time of 50ms. The acquisition was performed using Nth Order Double Play mode. Full scan profile mass spectra was acquired over a *m*/*z* of 400–2000 Da at a frequency of 1 spectrum every second. Top 10 intense ions were targeted for MS/MS under an isolation width of 2*m*/*z* units in CID mode with a collision energy of 35. Switching criteria were set to ions greater than mass to charge ratio (*m*/*z*) of 400 and smaller than *m*/*z* of 2000 with a charge state of 2–5. An abundance threshold of more than 500 counts and the target ions were excluded for 30 s with a repeat duration of 30 s and repeat count set to one. Duplicates of every fraction was injected and acquired.

## 3 Mass spectrometry data analysis

**Fig. 1:**
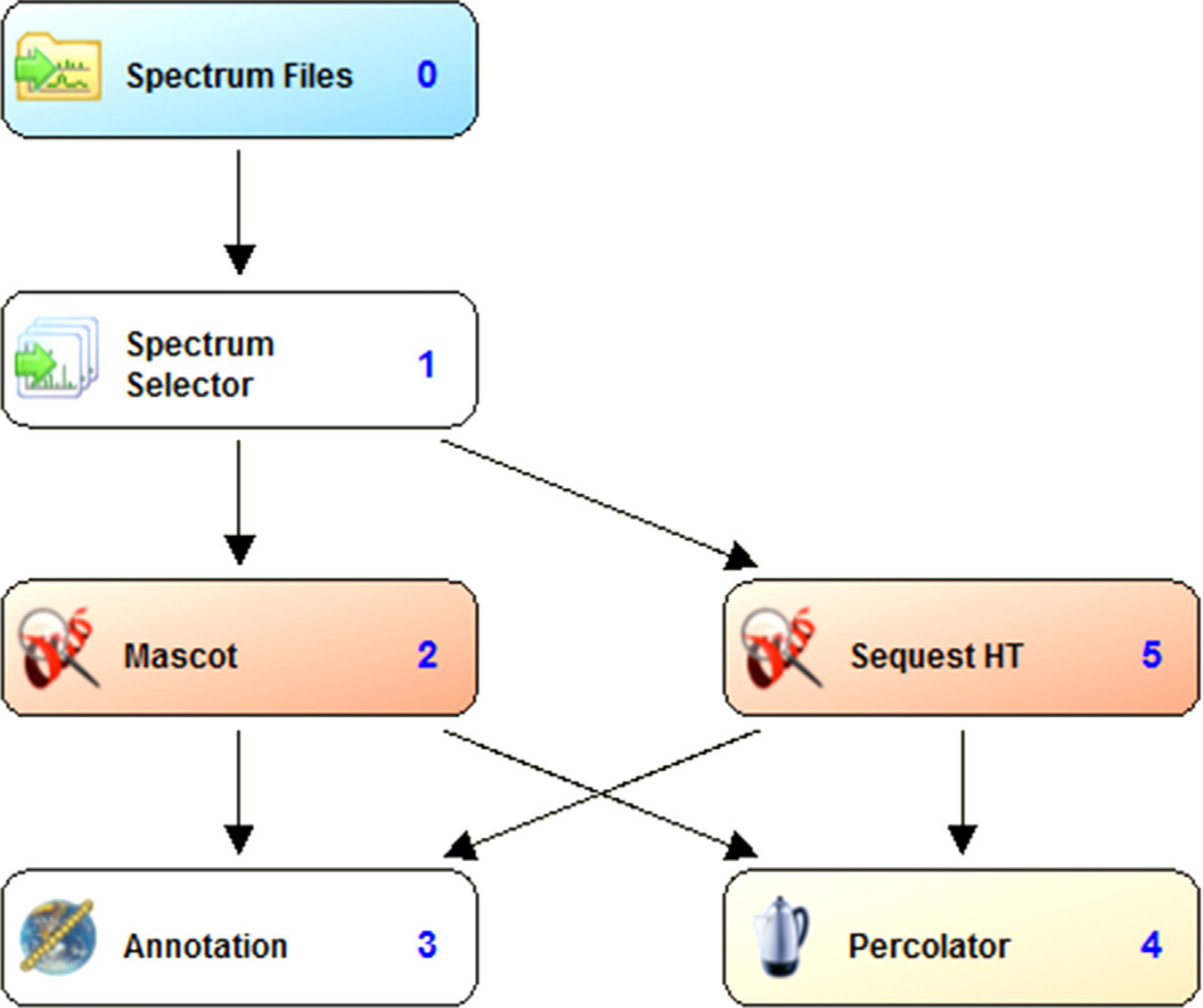
Workflow followed for protein identification using proteome discoverer. Raw files generated by the Orbitrap Velos Pro mass spectrometer were searched against the *A. flavus* protein sequences from Uniprot using two different search algorithms. The PSMs from each search result was subsequently validated by the Percolator algorithm. In addition, the annotations for each of the identified protein were retrieved from ProteinCenter.

**Fig. 2:**
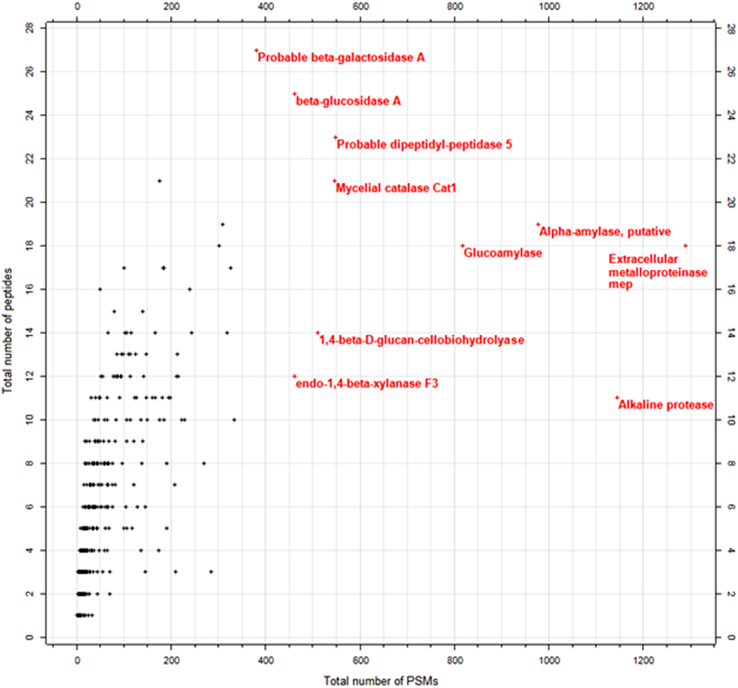
Correlation between the number of peptides and the PSMs for the identification of exoproteins. Every protein identified in the *A. flavus* exoproteome is plotted as a function of total number of peptides contributing to its identification (Y-axis) and the total number of PSMs (X-axis) that is an indicator of the protein abundance.

The workflow followed in the analysis and identification of proteins using Proteome Discoverer (PD) version 1.4.1.14 is shown in Fig. 1. Each of the raw files acquired from Orbitrap Velos Pro Mass Spectrometer were searched against the complete proteome of *Aspergillus flavus* (strain ATCC 200026 / FGSC A1120/NRRL 3357/JCM 12722/SRRC 167) including the isoforms downloaded from the Uniprot database on 30th August, 2013 (13501 entries). The PD workflow was built using spectrum selector node connected to Mascot and SequestHT search nodes. A peptide tolerance of 10 ppm was set for both nodes and a fragment tolerance of 0.60 Da and 0.80 Da was set for SequestHT and MASCOT, respectively. Two missed cleavages were allowed during the search. Cysteine carbamidomethylation was given as fixed modification while methionine oxidation, N-terminal acetylation and phosphorylation (S, T, Y) as variable modifications for both the nodes. Both the search nodes are connected to the Percolator node for PSM validation and the Annotation node that is connected to retrieve the Gene Ontology (GO) annotations for each protein from ProteinCenter. The FDR was set at 0.01 and the validation was based on the q-value in the percolator node [2,3]. All the.msf files were opened together as a single multi-consensus report with protein and peptide grouping enabled to generate a non-redundant list of identified proteins. The identifications were filtered using the result filters in PD tab by applying two filters to retain only the peptides identified with high confidence - peptide confidence was set to high and the peptide rank was set to 1. Summary of the protein identifications is given in Supplementary Table 1. A scatter plot of the total number of peptides against the total number of PSMs highlighting the top abundant proteins is shown in Fig. 2. The data have been deposited to the ProteomeXchange Consortium [4] via the PRIDE partner repository with the dataset identifier PXD001296.

### 3.1 Enrichment analysis of the identified proteins

**Fig. 3:**
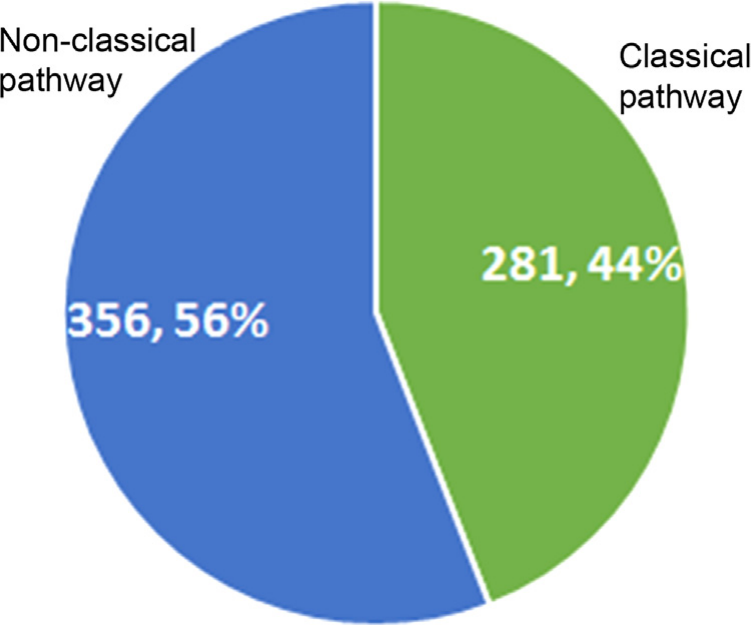
Distribution of proteins secreted through classical and non-classical pathways. Identified exoproteins were compared against the proteins predicted to be secreted through classical pathway to identify those proteins lacking a N-terminal signal sequence and secreted probably through non-classical pathway(s).

GO annotations of the identified proteins were used to determine the enrichment of specific functional categories in the exoproteome. Proteins belonging to the top five biological process categories (Supplementary Table 2) and molecular functions (Supplementary Table 3) provides an insight into the major functions of the exoproteins. The identified proteins were searched for the GO term “Peptidase activity” (GO: 0008233). And, in addition, the proteases of *A. flavus* that are cataloged in the MEROPS database [5] were also included to get a list of proteases secreted by *A. flavus* (Supplementary Table 4). Nearly 56% of the exoproteins identified in this study are probably secreted through the non-classical pathways (Fig. 3, Supplementary Table 5). Amongst the identified exoproteins, several proteins have been implicated in virulence in other fungal pathogens (Supplementary Table 6). Interestingly, many of these are proteases, which are found in high abundance (based on PSMs) in *A. flavus* exoproteome.

## Conflict of interest

The authors declare no conflict of interest pertaining to this research.
