# Supplementary material for: Data set for the mass spectrometry based exoproteome analysis of Aspergillus flavus isolates
Source: Data Brief. 2014 Dec 15;2:42–7. doi: 10.1016/j.dib.2014.12.001 (PMC4459775; doi:10.1016/j.dib.2014.12.001)
Supplement: Supplementary file 1 — Supplementary material [file mmc1.zip › Supplementary Table 1.docx]

| **Sample pooling details** | **Proteins identified with** | |
| --- | --- | --- |
|  | **≥ 1 peptide(s)** | **≥ 2 peptides** |
| 1 - 5 fractions | 213 | 127 |
| 6 -10 fractions | 339 | 214 |
| 11 -15 fractions | 409 | 233 |
| 16 - 20 fractions | 371 | 209 |
| 21 - 22 fractions | 80 | 17 |
| **Total number of proteins** | **637** | **368** |
