# Supplementary figures and images for: Data set for the mass spectrometry based exoproteome analysis of Aspergillus flavus isolates
Source: Data Brief. 2014 Dec 15;2:42–7. doi: 10.1016/j.dib.2014.12.001 (PMC4459775; doi:10.1016/j.dib.2014.12.001)

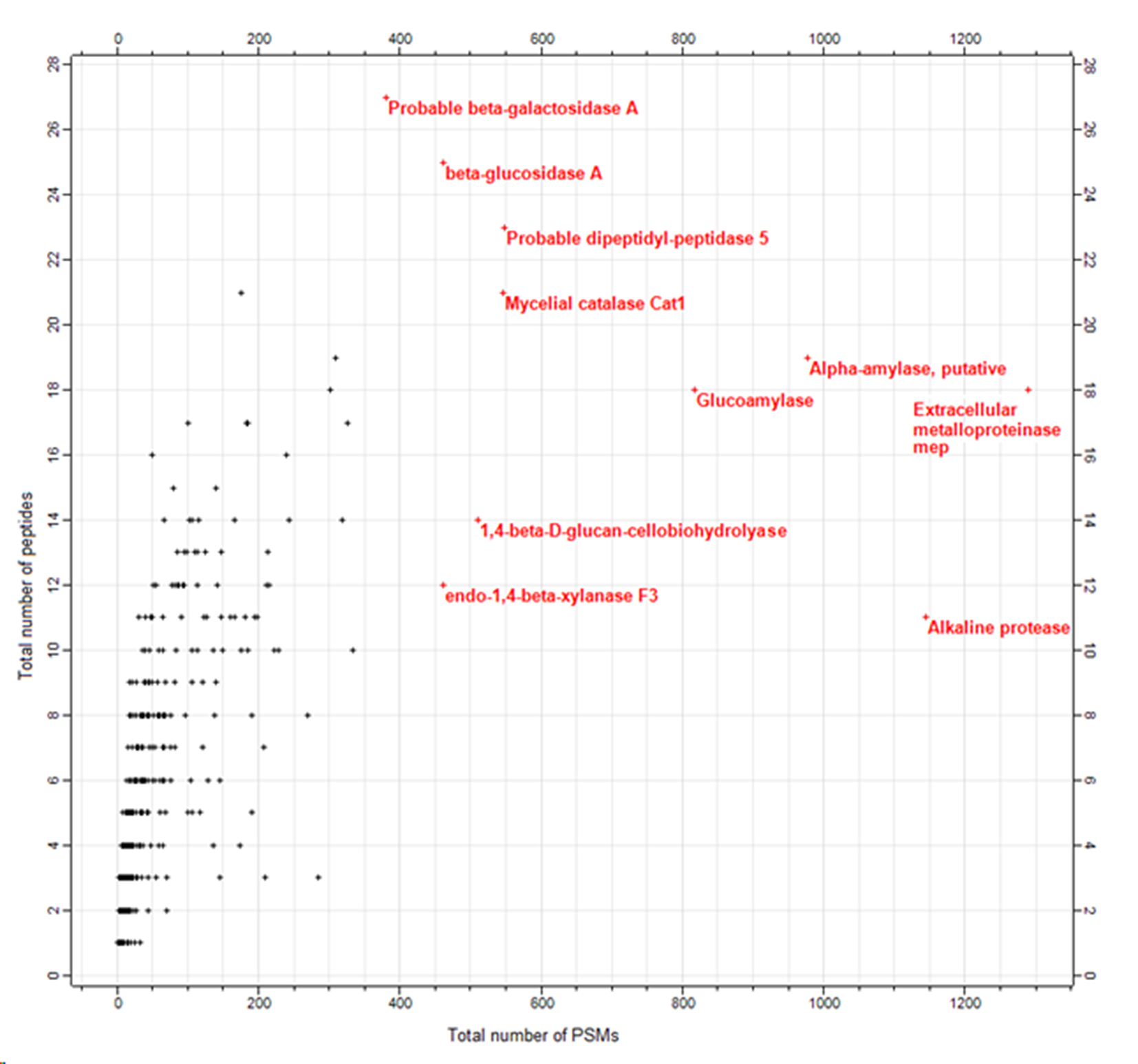

Supplement: Supplementary file 1 — Supplementary material [file mmc1.zip › Supplementary Figure 1.tif]
